# Supplementary material for: Identification of Freezing-Responsive microRNAs and Their Targets in Chinese Jujube by Small RNA and Degradome Sequencing
Source: Int J Mol Sci. 2026 Sep 4;27(17):7912. doi: 10.3390/ijms27177912 (PMC13566890; doi:10.3390/ijms27177912)
Supplement: Supplementary file 1 [file ijms-27-07912-s001.zip › Supplementary Material S2/novel_miR_212.pdf]

Provisional ID : novel_miR_212
Score total : 0.9
Score for star read(s) : -1.3
Score for read counts : 0
Score for mfe : 0.6
Score for randfold : 1.6
Score for cons. seed :
Total read count : 95
Mature read count : 95
Loop read count : 0
Star read count : 0

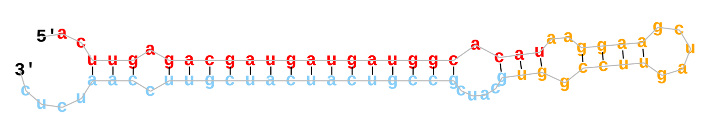

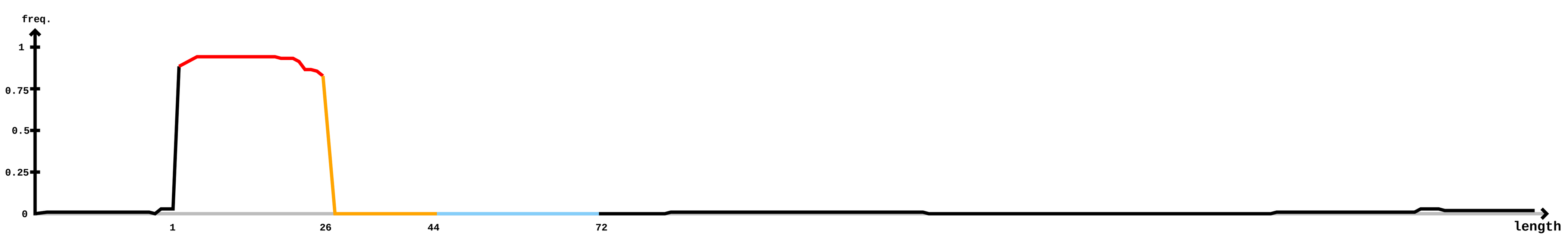

Mature Star

```
5'- augguacaagcucgaagccauuacuugagacgaugaugauggcacauaaggaagcuaguuccggugcaucgccgucaucaucguuccaaucucuauccaaauugguggaaaugaccauuguggacauugaugaccuacaaucucuuccacaagaagguguggccuaccucucaucucuucaaucucuucagauuagcaguugccccaauuugacgucauugccagaaucuaucggcaaccucucaucacu -3'   exp
    (((((..........)))))...(((.((((((((((((((.(((..((((.....)))).)))....)))))))))))))).))).......(((((((.((.(((..(..(((((((....(((........)))...)))))))(((((.((((........)))).)))))(((((....))))).)..))).))))))))))......(((((.((.....)))))))...........   reads   mm   sample
    augguacaagcucgaagc........................................................................................................................................................................................................................................   1   0   LCK-3
    ..................auuacuugGgacgaugauga....................................................................................................................................................................................................................   1   1   LT-1
    ..................auuacuugagacgaugaugaugg.................................................................................................................................................................................................................   2   0   LT-2
    ......................acuugGgacgaugaugauggc...............................................................................................................................................................................................................   2   1   LT-1
    ......................acuugagacgaugaugauggc...............................................................................................................................................................................................................   1   0   LT-1
    ......................acuugagacgaugCugauggcacau...........................................................................................................................................................................................................   1   1   LCK-2
    ......................acuugagacgaugaugauggcacau...........................................................................................................................................................................................................   44   0   LCK-1
    ......................acuugagacgaugaugauggcacau...........................................................................................................................................................................................................   41   0   LCK-2
    .......................cuugGgacgaugaugauggcaca............................................................................................................................................................................................................   2   1   LT-3
    ........................uugGgacgaugaugauggc...............................................................................................................................................................................................................   2   1   JCK-3
    .........................ugagacgaugaugauggcac.............................................................................................................................................................................................................   1   0   LT-3
    .........................ugGgacgaugaugauggcaca............................................................................................................................................................................................................   1   1   JCK-2
    ........................................................................................................guggaaaugaccauuUggaca.............................................................................................................................   1   1   LT-1
    ..............................................................................................................................uugaugaccuacaaucucuuc.......................................................................................................   1   0   JCK-3
    .............................................................................................................................................................................................................caauuugacgucauugccagaaucuauc.................   1   0   JCK-3
    .....................................................................................................................................................................................................................................uaucggcaaccucucaucacu   1   0   JCK-1
    .....................................................................................................................................................................................................................................uaucggcaaccucucaucacu   1   0   JCK-3
```
